# Three-dimensional hot electron photovoltaic device with vertically aligned TiO_2_ nanotubes

**DOI:** 10.1038/s41598-018-25335-6

**Published:** 2018-05-09

**Authors:** Kalyan C. Goddeti, Changhwan Lee, Young Keun Lee, Jeong Young Park

**Affiliations:** 1 Center for Nanomaterials and Chemical Reactions, Institute for Basic Science (IBS), Daejeon, 305-701 Korea; 2 Graduate School of EEWS, Korea Advanced Institute of Science and Technology (KAIST), Daejeon, 305-701 Korea

## Abstract

Titanium dioxide (TiO_2_) nanotubes with vertically aligned array structures show substantial advantages in solar cells as an electron transport material that offers a large surface area where charges travel linearly along the nanotubes. Integrating this one-dimensional semiconductor material with plasmonic metals to create a three-dimensional plasmonic nanodiode can influence solar energy conversion by utilizing the generated hot electrons. Here, we devised plasmonic Au/TiO_2_ and Ag/TiO_2_ nanodiode architectures composed of TiO_2_ nanotube arrays for enhanced photon absorption, and for the subsequent generation and capture of hot carriers. The photocurrents and incident photon to current conversion efficiencies (IPCE) were obtained as a function of photon energy for hot electron detection. We observed enhanced photocurrents and IPCE using the Ag/TiO_2_ nanodiode. The strong plasmonic peaks of the Au and Ag from the IPCE clearly indicate an enhancement of the hot electron flux resulting from the presence of surface plasmons. The calculated electric fields and the corresponding absorbances of the nanodiode using finite-difference time-domain simulation methods are also in good agreement with the experimental results. These results show a unique strategy of combining a hot electron photovoltaic device with a three-dimensional architecture, which has the clear advantages of maximizing light absorption and a metal–semiconductor interface area.

## Introduction

Plasmonic energy conversion has been studied extensively as an effective pathway for converting solar energy to current in comparison with typical electron–hole generation in semiconductor devices. Surface plasmons exhibit particular advantages for field concentration, light trapping, and the generation of hot carriers that provide a wide range of possible applications within the fields of photocatalysis and solar cells. Suitably designed nanostructures exhibit a localized surface plasmon resonance (LSPR) in which free electrons oscillate in resonance with the incident light, thus establishing electromagnetic fields that are highly localized. Furthermore, this efficient light trapping in plasmonic nanostructures can be coupled with semiconductor devices for photovoltaic applications^1,2^. Several recent studies have reported that plasmonic metal nanostructures can be utilized to convert collected light directly into electrical energy by generating electrons^3–10^. When LSPR excitation occurs in plasmonic nanostructures, the decay of the electromagnetic field happens very rapidly at timescales of femtoseconds; in particular, decay via non-radioactive processes proceeds by the transfer of energy to free hot electrons^9,11–14^. These hot electrons are not in thermal equilibrium with the atoms in the lattice and are often characterized by the Fermi function with elevated temperatures^15–17^. Furthermore the excitation of hot electrons can be observed in dye-sensitized solar cells during exothermic chemical processes^18^. In these solar cells, a dye molecule that is functionalized to the semiconductor absorbs the incoming solar energy and the transfers the energetic hot carriers to the semiconductor. This strategy is limited to the utilization of specific light wavelengths by unstable anchoring of the molecule, which causes poor stability and durability of the system. Plasmonic nanostructures can transfer energy from 1 to 4 eV; this energy directly affects carrier concentration and the shape of the nanostructures^7,19^. Y.K. Lee *et al*. proposed the fabrication of a Schottky diode with Au/TiO_2_ nanostructures to efficiently probe hot electron flow enhanced by surface plasmons in Au connected island structures^20^. Enhancement of internal photoemission through surface plasmons was also investigated with Ag/TiO_2_ where a thin Ag film (the plasmonic material) was deposited on TiO_2_, thus forming metal–semiconductor contacts. The Ag film showed a higher incident photon to current conversion efficiency (IPCE) when compared with a Au film on TiO_2_. This enhancement was attributed to surface plasmons originating from the corrugated Ag surface. Consequently, further detailed studies on the effects of thickness and morphology were conducted to observe the effect of plasmonic behavior on connected island structures^21^. The enhanced hot electron flux on various schemes adapting nanowire^22^, dye molecules^23^, or stacked tandem structures^24^ were demonstrated. Furthermore, C. Lee *et al*. demonstrated an effective way to utilize plasmon-induced hot electrons with minimal losses by employing a metal–insulator–metal heterojunction. The tunneling phenomenon is predominant in these junctions, where lower-energy excited electrons can be captured by tunneling through the insulator to the other side of the metal^25^.

In this study, we report the electrochemical preparation of titanium dioxide nanotubes (TNA) with various porosities by controlling the voltage during anodization as well as the fabrication of a Schottky diode to investigate the dependence of plasmonic behavior on the size and shape of the plasmonic material in the visible region for hot electron generation. In general, TiO_2_ has been an extensively investigated material since the discovery of the photolysis of water using a single crystal of TiO_2_ by Fujishima and Honda^26^. The use of ordered one-dimensional nanotubular structures has been demonstrated in solar cells^27,28^, sensors^29,30^, H_2_ evolution by water splitting^31–33^, Li-ion batteries^34,35^, supercapacitors^36^, and drug delivery applications^37^. Several potential devices using nanostructures to create new efficient environmentally friendly devices have been demonstrated; the development of large-scale, easy-to-manufacture, simple, and cost effective nanoarchitectural devices are always in demand, especially for energy conversion/storage applications^38,39^. Green nanoarchitectures, including hybrid assemblies of zero-dimensional and one-dimensional nanomaterial devices, created using simple and efficient fabrication techniques have been reported for applications in energy conversion and storage^40–42^. The one-dimensional nanostructure exhibits fascinating properties and attractive performance because of its large surface area upon which various chemical reactions occur and the direct orthogonal electron path through the tubular walls that results in efficient charge transport in photoanodes^43^. Despite these motivating properties, in general, the primary drawback of TiO_2_ is its transparency in visible light. Band gap engineering of photoactive materials has been attempted using various strategies, including metal or non-metal doping^44,45^, metal deposition^46,47^, and heterocoupling with narrow-band gap semiconductors^48,49^. Coupling a semiconductor with a surface plasmonic material is an efficient method to harvest the abundant low-energy photons. Plasmonic nanostructures act as an antenna to capture the incoming light with minimal reflection. These plasmonic structures generate hot electrons through the decay process that occurs transverse across the semiconductor material, thus contributing to the photocurrent.

Using optimized parameters, we synthesized nanotubes by anodizing e-beam deposited Ti on a SiO_2_ substrate followed by deposition of a thin layer (35 nm) of a plasmonic metal (Au or Ag) on the nanotubes, thus forming three-dimensional Au/TNA or Ag/TNA Schottky diodes. From the IPCE measurements, we clearly observed the effect of surface plasmons on the photocurrent. Plasmonic/one-dimensional semiconductor nanodiodes can be used for solar energy conversion as an alternative to dye-sensitized solar cells. Further improvements of the diode efficiency can be attained to explore the fields of solar cells and photocatalysis.

## Results and Discussion

**Figure 1:**
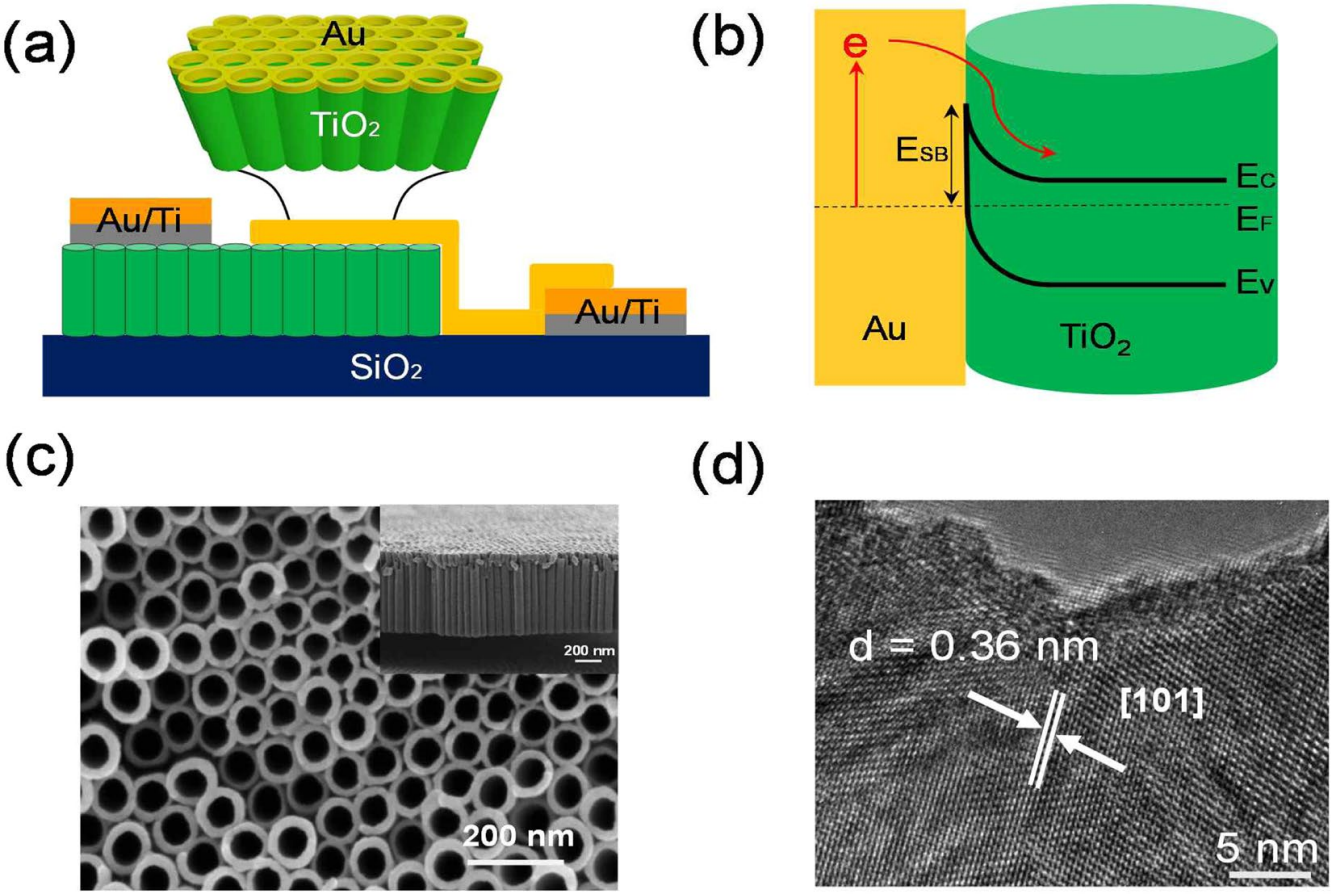
(**a**) Schematic of the plasmonic nanodiode based on TiO_2_ nanotubes with a 35 nm thick active layer of Au. (**b**) Energy band diagram of the Schottky junction depicting hot electron generation following photo excitation. (**c**) Scanning electron microscopy images of the TiO_2_ nanotubes fabricated using anodization and then annealed at 450 °C for 2 hr in air. Inset shows a side view of the nanotubes. (**d**) High-resolution transmission electron microscope image of the anatase [101] TiO_2_ nanotube wall surface showing an inter-planar distance of 0.36 nm.

TNA with various pore diameters were synthesized via a two-step anodization process. The plasmonic metal deposited on these structures forms plasmonic Au (or Ag)/TNA diodes with different sizes. Figure 1a depicts the Au/TNA Schottky diode schematic and the energy band diagram of the Schottky junction of Au on TiO_2_ is illustrated in Fig. 1b. The interface of the Au and TiO_2_ is the active area of the diode; hot electrons can be generated in the plasmonic Au and these hot electrons can overcome the Schottky barrier (*E*_*SB*_) and reach to the semiconductor material. Decay of the plasmonic mode can occur via either a radiative (scattering) process or a non-radiative (absorption) process. During the non-radiative decay process, plasmonic energy is initially transferred to a lone hot electron–hole pair. However, when a plasmonic nanostructure is joined with a semiconductor, thus establishing a Schottky barrier (*E*_*SB*_) potential at the interface, hot electrons can traverse the Schottky barrier when they have sufficient momentum instead of rapidly thermalizing with the surrounding electron gas^9,20^. Scanning electron microscopy (SEM) images of TiO_2_ nanotubes are shown in Fig. 1c (top view) and the corresponding side view image is depicted in the inset in Fig. 1c. A thin (35 nm) plasmonic gold layer was deposited on the anodized TNA structures. The Au (or Ag) on TNA represents the active area of the nanodiode where hot electron excitation takes place and overcomes the Schottky barrier. Figure S2 shows a SEM image of the TiO_2_ nanotubes with a 35 nm thick layer of plasmonic Ag metal. After deposition of the metal layer on the TiO_2_ nanotubes, we found that the metal layer constitutes a network of thin layered plasmonic metal on nanotubes that allows electrical contact within the diode.

The crystallinity of the annealed nanotubes was measured using transmission electron microscope (TEM) images of the nanotubes. An individual nanotube can be separated from the substrate by either sonication or immersion in hydrochloric acid; the individual nanotubes were later dispersed in ethanol for characterization. An interplanar distance of 0.36 nm was measured from the high-resolution image of the nanotube wall shown in Fig. 1d. The as-synthesized nanotubes are typically amorphous in nature and highly insulating. After annealing the samples in a muffle furnace at 450 °C for 2 hours (heating rate of 1 °C/min), we obtained the anatase phase of TiO_2_, which was confirmed from the XRD peaks. Figure S3 shows X-ray diffraction (XRD) measurements of the Au/TNA to measure the crystalline properties of the TNA and the Au.

**Figure 2:**
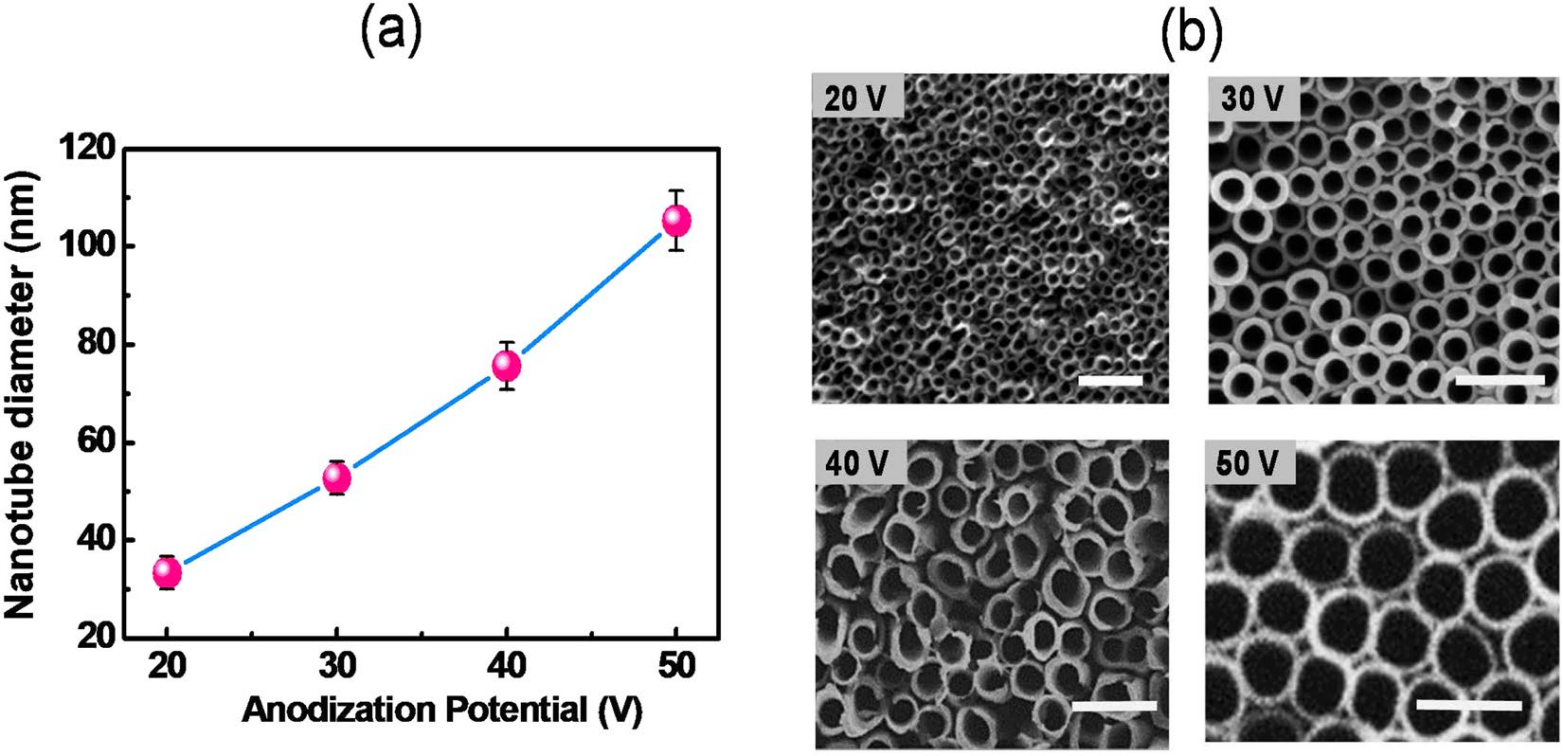
(**a**) Plot of nanotube pore diameter dependence as a function of applied potential during the anodization process. (**b**) Corresponding SEM images of the TiO_2_ nanotubes fabricated using voltages of 20–50 V. Scale bar: 200 nm.

Typically, plasmonic nanostructures with different sizes and shapes can exhibit various plasmonic resonances at different wavelengths of the visible spectrum. This phenomenon helps us to efficiently harvest visible light energy. Here, we synthesized TNA with diverse porosities by changing the anodization potentials, and then Au (or Ag) was deposited on the TNA, thus creating plasmonic nanostructured systems with different porosities. Figure 2a shows a plot of the nanotube diameter versus the applied DC potential during the anodization process. We can clearly observe that the diameter of the tubes is proportional to the applied potential. In general, smaller potentials initiate small pit centers on the Ti surface during the initial anodization stages. These centers then serve as nucleation sites and further dissolution of the Ti beneath the pits creates a pore with voids between adjacent nanotubes. Thus, the potential affects both the pore size and the wall thickness of the nanotubes. This trend is indicated in Fig. 2b in which the size of the nanotube diameter increases from 33 nm (at 20 V) to 105 nm (at 50 V). Figure S1 provides size distribution histograms of the nanotubes. The optical properties of the Au/TNA were observed by measuring the absorbance in the UV-Vis-NIR region (300–800 nm). Figure S4 shows a plot of the absorbance spectrum of the Au/TNA in which a sharp increase in the UV region corresponds to optical excitation of the TiO_2_ and the peak in the visible region (observed at 580 nm for Au/TNA 30 and at 600 nm for Au/TNA 50) represents the surface plasmons of the Au nanostructure deposited on the TNA.

**Figure 3:**
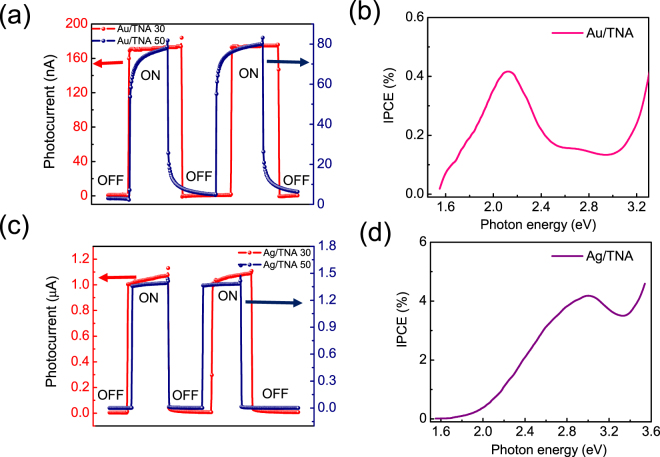
(**a**) Photocurrent measurements of the Au/TNA 30 and Au/TNA 50 nanodiodes by exciting the diode with a light source during a period of on/off conditions. (**b**) Incident photon to current conversion efficiency (IPCE) measurements as a function of photon energy for the Au/TNA with the peak observed at 584 nm (2.12 eV) corresponding to the surface plasmonic peak of the active Au thin layer on TiO_2_. (**c**) Photocurrent measurements of the Ag/TNA nanodiodes. The Ag/TNA 30 nanodiode has a photocurrent of 1.1 µA and the photocurrent for the Ag/TNA 50 nanodiode is around 1.4 µA. (**d**) IPCE measurements as a function of photon energy for the Ag/TNA with the peak observed at 414 nm (2.99 eV) corresponding to the surface plasmonic peak of the active Ag thin layer onTiO_2_.

The electrical properties of the Au/TNA and Ag/TNA nanodiodes were measured using current–voltage curves by sweeping the voltage across the electrodes from −1 to +1 V. The rectifying behavior of the diode was clearly observed in Figure S5 (inset). We calculated the Schottky barrier heights and ideality factors of the diodes by fitting the current–voltage curves to the thermionic emission equation, as shown in Figure S5^50^. Figure S5a–d represents thermionic emission fitting of the I–V results from the nanodiodes. Schottky barrier height values of 0.78 and 0.67 eV were calculated for the Ag/TNA 50 and for Ag/TNA 30 nanodiodes, respectively. Similarly, the barrier heights for the Au/TNA 50 (0.73 eV) and Au/TNA 30 (0.76 eV) were also obtained after fitting the respective I–V curves. In general, the Schottky barrier height of the Au/TiO_2_ can be determined by the difference between the work function of gold (around 5 eV) and the electron affinity of TiO_2_ (4–4.2 eV). Thus, the barrier height corresponds to 0.8–1 eV, which is within the range of values we obtained from thermionic emission fitting. Figure 3a shows the short-circuit photocurrent measurements of the Au/TNA diode obtained by the illumination using a tungsten-halogen lamp with an incidence angle to the plane of the diode during a period of on/off conditions. An efficient photocurrent of around 160 nA was derived from the Au/TNA 30 nanodiode, which clearly shows a significant three-fold enhancement of the device when compared with thin film nanodiodes^20^. Likewise, a photocurrent of 80 nA was observed with the Au/TNA 50 nanodiode, a two-fold enhancement in the photocurrent.

Figure 3b shows the IPCE measurements performed to obtain the conversion efficiency as a function of photon energy from the Au/TNA diode. IPCE can provide information correlating surface plasmons and the generation of hot electrons as originating in the short-circuit photocurrent of the Au/TNA diode. We observed a plasmonic peak at 2.12 eV (584 nm) for the Au/TNA diode. This plasmonic excitation is in good agreement with the optical properties of the Au/TNA 30 and Au/TNA 50 (shown in Figure S4) where the absorbances were 580 and 600 nm, respectively. This clearly indicates that the excitation of Au at these wavelengths induced the generation of hot electrons. Figure 3c represents the photocurrent and IPCE of the Ag/TNA nanodiodes. Photocurrents were obtained and the Ag/TNA 50 had an outstanding photocurrent of 1.4 µA. The plasmon peaks from IPCE were obtained at higher energies of 2.99 eV (414 nm), as seen in Fig. 3d. The higher IPCE of 4% was achieved with the Ag/TNA diode compared with the Au/TNA diodes, which explains the phenomenon of Ag having enhanced near fields compared with Au. Implementation of one-dimensional nanotubular structures for efficient charge carrier transport and the intrinsic higher near fields of Ag than Au results in a significant increase in both photocurrent and IPCE.

At lower photon energies, the effect of TiO_2_ can be completely ruled out because of its wide band gap (3.2 eV). At higher photon energies (*hv* > 3.2 eV), excitation of electron–hole pairs occurs in in the TiO_2_, resulting in the IPCE increasing significantly above 3 eV in addition to internal photoemission^51^. When external energy is induced (via photon energy or chemical reactions) on the metal surface, hot electrons can be generated non-adiabatically with energies above the fermi level. Thus, the vital mechanism for various energy conversion applications can be directed by the dynamics of these generated hot electrons. When the source of the external energy is photons, the efficiency of the device primarily depends on the optical properties of the photon absorber. Utilization of plasmonic materials can be one such surface modification to allow efficient light trapping and to enhance internal photoemission. During its decay process, the excited localized surface plasmons in the plasmonic metal amplify hot electron flow. The Schottky barrier is fundamentally crucial for extracting the energetic hot electrons by reducing the plasmonic metal work function. Excited hot electrons from the plasmonic metal can effectively overcome the barrier height and reach the semiconductor surface on the other side before they relax as heat^52^. Au/TNA and Ag/TNA plasmonic nanodiodes were utilized to measure the steady-state currents from ballistic charge carriers that are generated in the plasmonic Au (or Ag) by surface plasmons. Table 1 compares the conversion efficiencies and short-circuit photocurrent studies of various plasmonic nanodiode systems with different architectures and metal thicknesses. From these references, we observed that Ag/TNA nanodiode systems have superior results compared with other nanodiode systeme, while there is still room for improvement in the case of Au/TNA. It is anticipated that efficiencies can be drastically changed by the thickness of the plasmonic metal.

**Table 1:** Comparision of various plasmonic nanodiode systems with TNA-based nanodiodes in terms of quantum efficiencies and short-circuit photocurrents.

Nanodiode System	Plasmonic metal thickness (nm)	Short-circuit Photocurrent (nA)	Reference
Au island/TiO_2_	10	45	^20^
Plasmonic Au/TiO_2_/Ti	10	80	^25^
Ag/TiO_2_	10	800	^21^
Ag/TiO_2_	30	300	^21^
Au/TNA	35	160	Current work
Ag/TNA	35	1400	Current work

**Figure 4:**
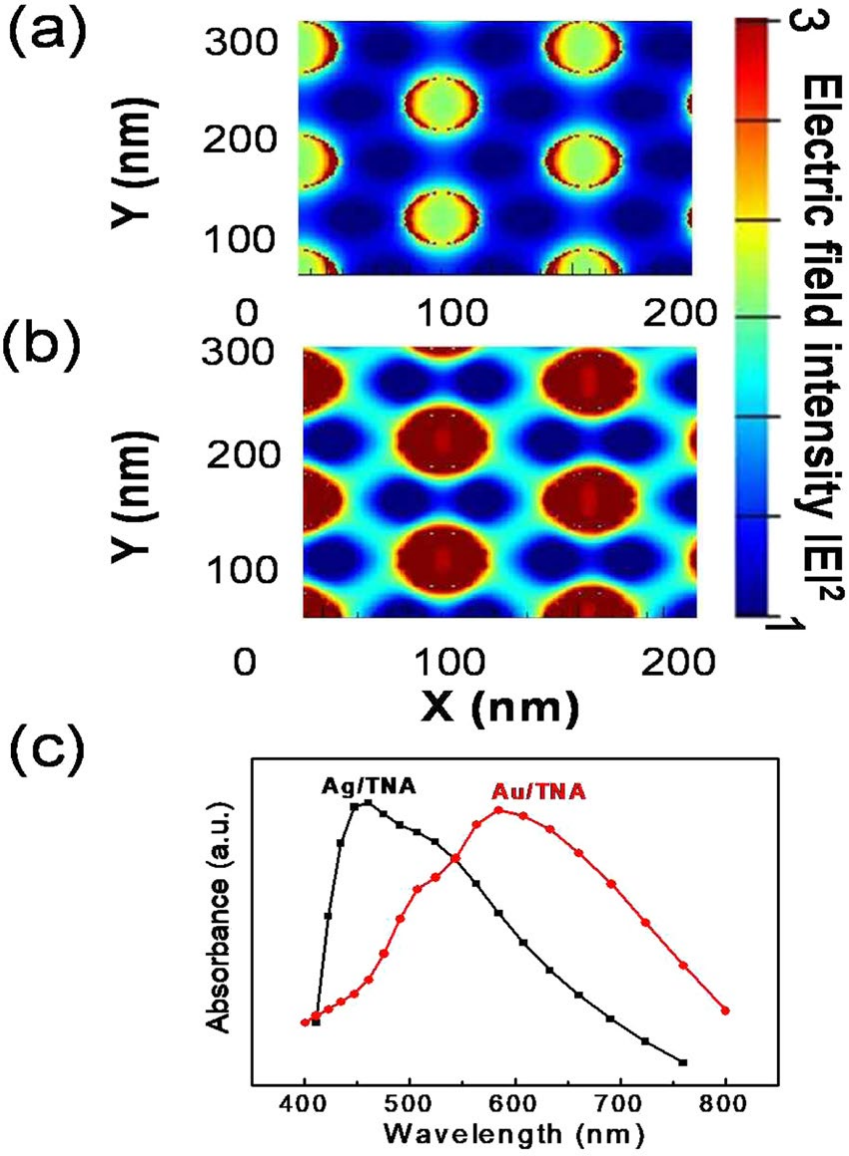
Finite-difference time-domain (FDTD) simulations: Calculated electric field distribution around patterned metal deposited on TiO_2_ nanotube of (**a**) Au/TNA and (**b**) Ag/TNA. (**c**) Calculated absorbance spectrum for nanohole-structured plasmonic metal deposited on TiO_2_ nanotube. The plasmonic peak was absorbed at 585 and 455 nm for Au and Ag, respectively.

To further understand the enhancement of photocurrent measured in these experiments, we carried out finite-difference time-domain (FDTD) simulations for the nanostructure with patterned nanohole Au and Ag deposited on a TiO_2_ nanotube under incident light at 582 nm (2.13 eV) and 451 nm (2.75 eV), respectively. The simulation results in Fig. 4a,b show the electric field distribution of the Au/TNA and Ag/TNA nanostructured architectures. The calculated electric field distribution indicates an enhancement of light absorption. A strong electric field is formed around the edge of the patterned metal nanohole structure. Note that the electric field is formed widely at the interface between the plasmonic metal and the TiO_2_. Figure 4c provides the calculated absorbance of the patterned Au and Ag nanohole structure on top of the TiO_2_ nanotube arrays. The peaks of the absorbance were obtained at 582 and 451 nm for Au and Ag, respectively, which provides strong support for the plasmonic peak generated during the IPCE measurements. For a more detailed comparison, simulations were carried out for the Au/TNA at various wavelengths that are outside of the effect of surface plasmon resonance. Figure S6a,b shows the electric field distributions at 450 nm (2.75 eV) and 750 nm (1.65 eV), respectively. The intensities of the electric fields at 450 and 750 nm are much weaker than that at 580 nm. The calculated results are in good agreement with the experimentally measured IPCE data, where the maximum was attained at circa 584 nm (2.12 eV), indicating an enhancement of light absorption by surface plasmon resonance. Thus, the surface plasmon activity was confirmed at 584 nm (2.12 eV) from the absorbance measurement where the peak in the IPCE plot was observed, implying that the majority of the current is due to hot electron generation induced by surface plasmons. Plasmonic nanodiodes with vertical TiO_2_ nanotubes can lead to advanced photovoltaic devices with high efficiency for hot electron conversion.

## Conclusions

In conclusion, we fabricated three-dimensional plasmonic nanodiodes using Au/TiO_2_ and Ag/TiO_2_ to investigate hot electron generation induced by surface plasmons for solar energy conversion. An anodization technique was used to fabricate and control the pore diameter of the nanotubes. The rectifying behavior of the diode was confirmed from I–V characteristics and used to calculate the ideality factor, series resistance, and Schottky barrier height by fitting the I–V curve with the thermionic emission equation. We obtained a barrier height of about 0.7 eV. The IPCE measurements confirmed plasmon-induced hot electron generation at lower photon energies (*hv* < 3 eV). The enhancement of IPCE observed at 2.99 eV is attributed to hot electrons generated by plasmonic Ag on TNA. Similarly, the peak at 2.12 eV is attributed to hot electrons generated by plasmonic Au on TNA. This was confirmed by the FDTD calculated absorbance of Au/TNA at 2.13 eV, which is in proximity of the peak obtained from the IPCE. These observations represent an enhancement of photocurrent on the Au/TNA nanodiode predominantly from plasmon-induced hot electrons. The higher photocurrent and IPCE of Ag is mainly from efficient charge carrier transport through the walls of the TiO_2_ nanotubes and the intrinsically higher near fields of Ag than Au metal. Due to the ease of fabrication, large surface area, and stability, plasmonic diodes based on nanotube array can open a window for efficient and practical applications in solar energy conversion.

## Methodology

### Fabrication of TiO_2_ nanotubes (TNA)

All of the reagents were analytical grade and used without further purification. Ammonium fluoride (NH_4_F, Junsei 97%), ethylene glycol (C_2_H_6_O_2_, Daejung 99%), isopropyl alcohol (C_3_H_8_O, Daejung 99.5%), ethanol (CH_5_OH, Merck), hydrochloric acid (HCl, Junsei), and DI water were used for anodization. The TNAs were prepared by anodizing Ti thin films deposited on a thermally oxidized SiO_2_ wafer substrate using an E-beam evaporator with a Ti tablet (99.99% pure). Prior to deposition, the SiO_2_ wafer was cleaned with ethanol and dried in flowing nitrogen gas. Two-step anodization was conducted in a typical two-electrode system with stainless steel (SUS 316) as the cathode and the e-beam deposited Ti on SiO_2_ as the anode. The anodization electrolyte consists of an ethylene glycol solution with 0.3 wt% of NH_4_F and 2 vol % of DI water^53^. The referred electrolyte is considered to be the optimized concentration for the growth of ordered and controlled nanostructures. The widely accepted growth mechanism of nanotubes is the near-simultaneous formation and subsequent dissolution of a barrier oxide layer with assistance from the electric field. Pore growth advances from the bottom of the pore towards the oxide–metal interface, thus organizing the nanotube structures. The net reactions for the field-assisted oxidation of titanium and the chemical dissolution of TiO_2_ in the fluoride electrolyte can be broadly summarized by the following equations (1) and (2)^54^

1 $${\text{Ti}+\text{xO}}_{2}\to {\text{TiOx}+\text{2xe}}^{-}$$

2 $$\text{TiOx}+\text{6F}\to {{{\rm{TiF}}}_{6}}^{2-}$$

Ammonium fluoride and DI water are the respective sources of fluorine and oxygen, with ethylene glycol being the conductive organic solution. Figure S1 shows the size distribution of the nanotubes formed using anodized Ti foil at 20–50 V. We prepared nanotubes with different pore sizes using conventional potentiostatic anodization. The parameters (dc voltage, electrolyte concentration, and time of the reaction) control the size, diameter, morphology, and the wall thickness of the nanotubes^55,56^. To fabricate the nanodiodes, a two-step anodization technique was employed to form a continuous nanotube array and to reduce crack formation during anodization. This process also forms a Schottky barrier without any short-circuits during the final steps of the nanodiode fabrication. Anodization of the Ti film with potentials of 30 and 50 V obtained nanotubes with increased diameters that are denoted as TNA 30 and TNA 50, respectively. After the first anodization step at the stipulated time periods for the individual voltages, the first anodized tubes were removed by immersing the samples in 1 M HCl for one hour and sonicating for 10 minutes to remove any residual debris from the tubes. The second anodization step was conducted with the same electrolyte as that used for the first anodization step, with time periods dependent on the voltages used. The as-synthesized samples are amorphous in nature and highly insulating. The amorphous phase can be transformed to a crystalline semiconductor by annealing the TNAs at 450 °C for 2 hours.

### Fabrication of Au/TNA Schottky diode

The first step in the diode fabrication starts with the deposition of electrodes on the annealed TNA 30 and TNA 50 structures. A 50 nm Ti layer was initially deposited to serve as the adhering layer for the gold as well as to obtain an ohmic contact between the TNA and the thick gold electrode. Next, a 150 nm thick gold film was deposited using the same mask as utilized for the initial Ti deposition step. This two-step process completes the electrode fabrication. Finally, a thin 35 nm layer of the plasmonic metal (Au or Ag) was deposited through a second mask onto the TNA structures, thus connecting the electrodes and constituting the active plasmonic layer.

### Measurements and Characterization

The size, morphology, and pore diameters of the nanotubes were characterized using field-emission scanning electron microscopy (FE-SEM, Magellan400). The annealed structures were analyzed using Cu-Kα X-ray diffraction with 2θ angle diffraction (RIGAKU, D/MAX-2500). The optical properties of the Au/TNA and Ag/TNA structures were observed at light wavelengths of 300–800 nm (Perkin Elmer, Lambda 1050) to study changes in the plasmonic peaks in the visible light region based on the size of the nanotubes. TEM images were obtained from a FEI Tecnai F30 ST transmission electron microscope at an accelerating voltage of 300 kV. The electrical measurements, such as the I–V characteristics, were conducted using a Keithley 2400 and the thermionic emission results were fitted to determine the Schottky barrier height of the diode. A tungsten-halogen lamp was used to obtain the photocurrents in the nanodiodes. The IPCE measurements were conducted using Oriel instruments (50–500 W power) within the visible spectrum. To further understand the distribution of electric fields and the enhancement of photocurrent measured in the experiments, we performed FDTD simulations of the nanostructures with patterned nanohole Au on TiO_2_ nanotubes as well as Ag on TNAs.

## Electronic supplementary material

Supplementary information
