# Supplementary material for: Three-dimensional hot electron photovoltaic device with vertically aligned TiO2 nanotubes
Source: Sci Rep. 2018 May 9;8:7330. doi: 10.1038/s41598-018-25335-6 (PMC5943325; doi:10.1038/s41598-018-25335-6)
Supplement: Supplementary file 1 — Supplementary information [file 41598_2018_25335_MOESM1_ESM.pdf]

Supplemental Information

Three-dimensional hot electron photovoltaic device with vertically aligned TiO_2 nanotubes

Kalyan C. Goddeti [a,b], Changhwan Lee [a,b], Young Keun Lee [a,b], and Jeong Young Park * [a,b]

[a]*Center for Nanomaterials and Chemical Reactions, Institute for Basic Science(IBS), Daejeon 305-701, Korea*

[b]*Graduate School of EEWS, Korea Advanced Institute of Science and Technology(KAIST), Daejeon 305-701, Korea*

* Correspondence and requests for materials should be addressed to J.Y.P (jeongypark@kaist.ac.kr)

KEYWORDS: TiO_2 nanotubes, anodization, surface plasmons, hot electrons, incident photon to current conversion efficiency (IPCE).

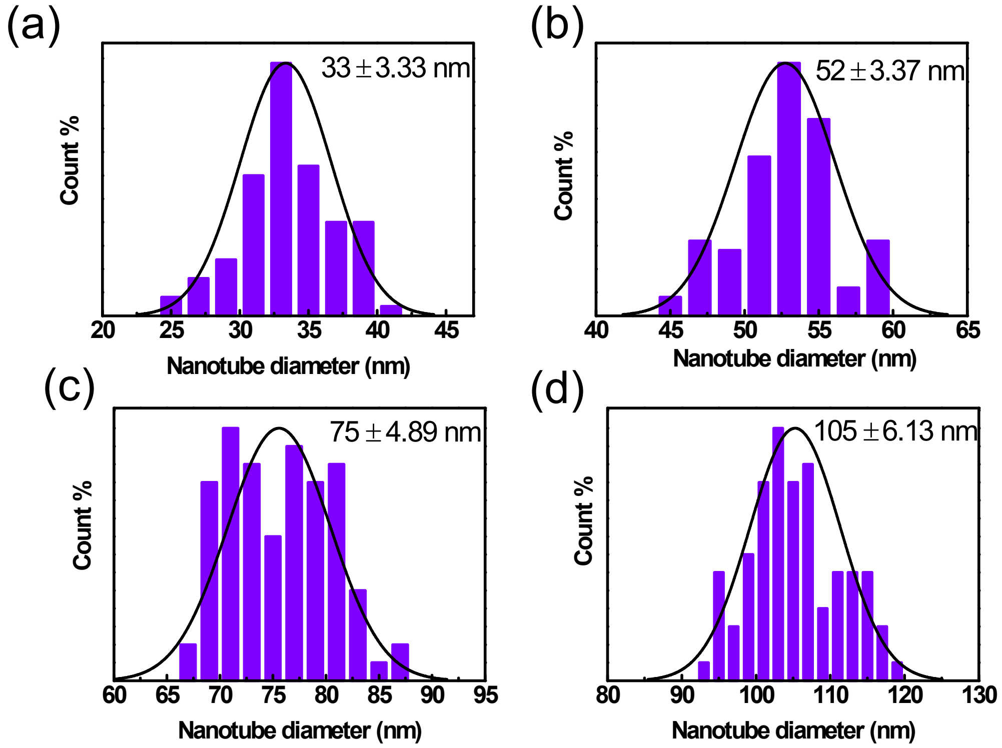

Figure S1. Size distribution histograms of the TiO_2 nanotube diameters, fabricated by anodizing at various potentials of **(a)** 20 V, **(b)** 30 V, **(c)** 40 V, and **(d)** 50V. The average diameters of the nanotubes with the standard deviations are shown.

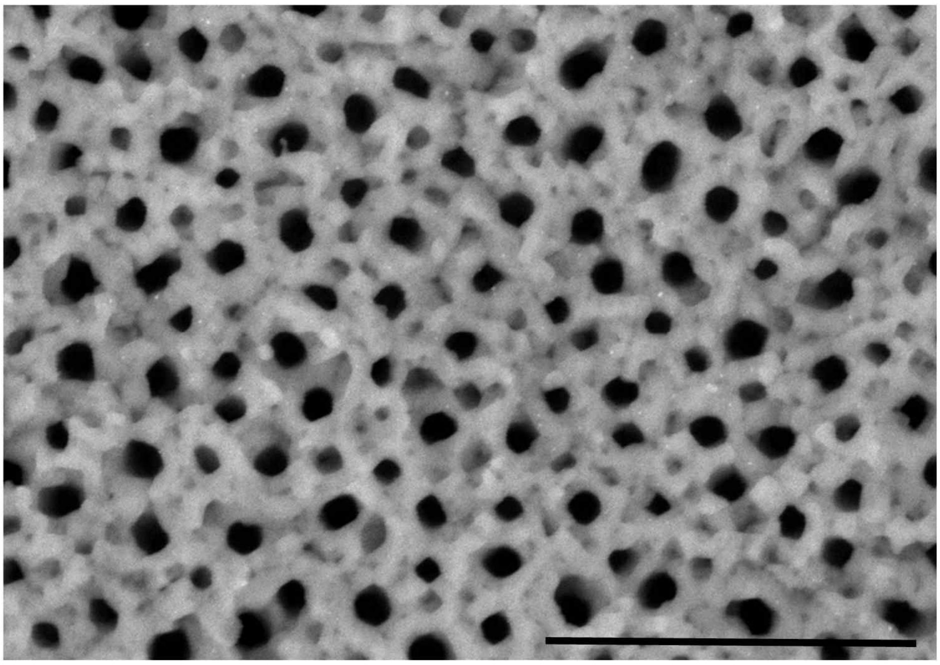

Figure S2. Scanning electron microscopy image of the Ag/TNA nanodiode with the deposition of 35 nm of the plasmonic metal on TiO_2 nanotubes to establish the active area of the nanodiode. Scale bar: 500 nm

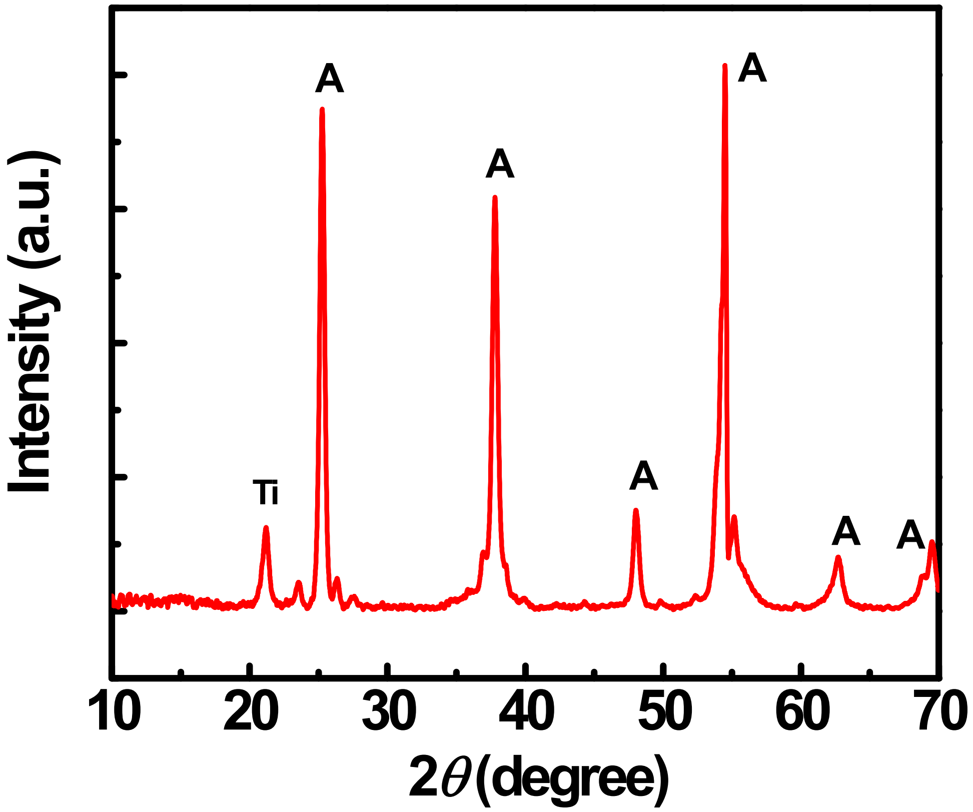

Figure S3. X-ray diffraction of the annealed TiO_2 nanotubes. The as-synthesized amorphous TiO_2 was completely crystallized to the anatase phase after annealing at 450° C for 2 hours. (A: anatase; Ti: titanium).

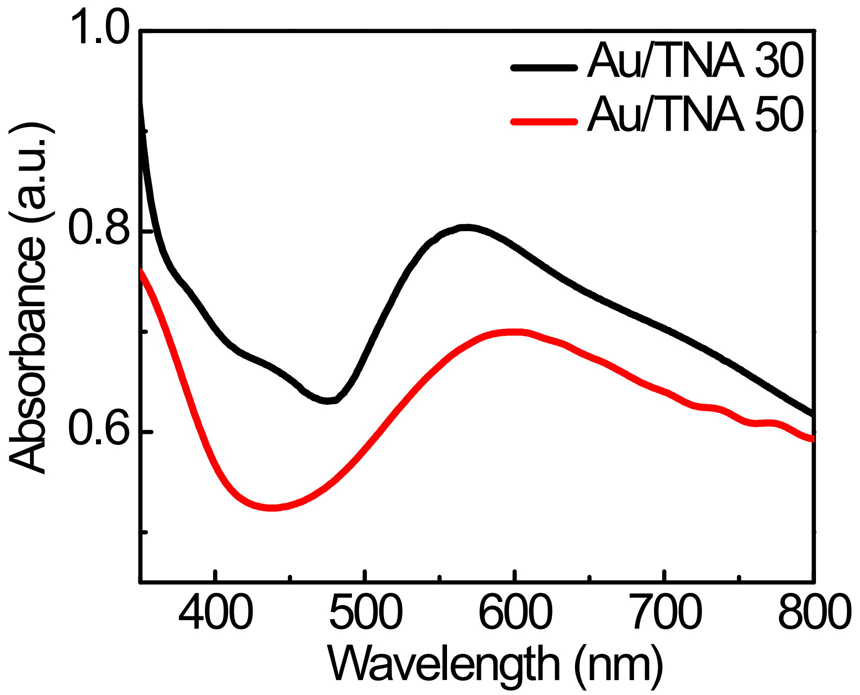

Figure S4. UV-Vis absorbance properties of the nanodiodes with the corresponding absorbances of 580 and 600 nm for the Au/TNA 30 and Au/TNA 50, respectively.

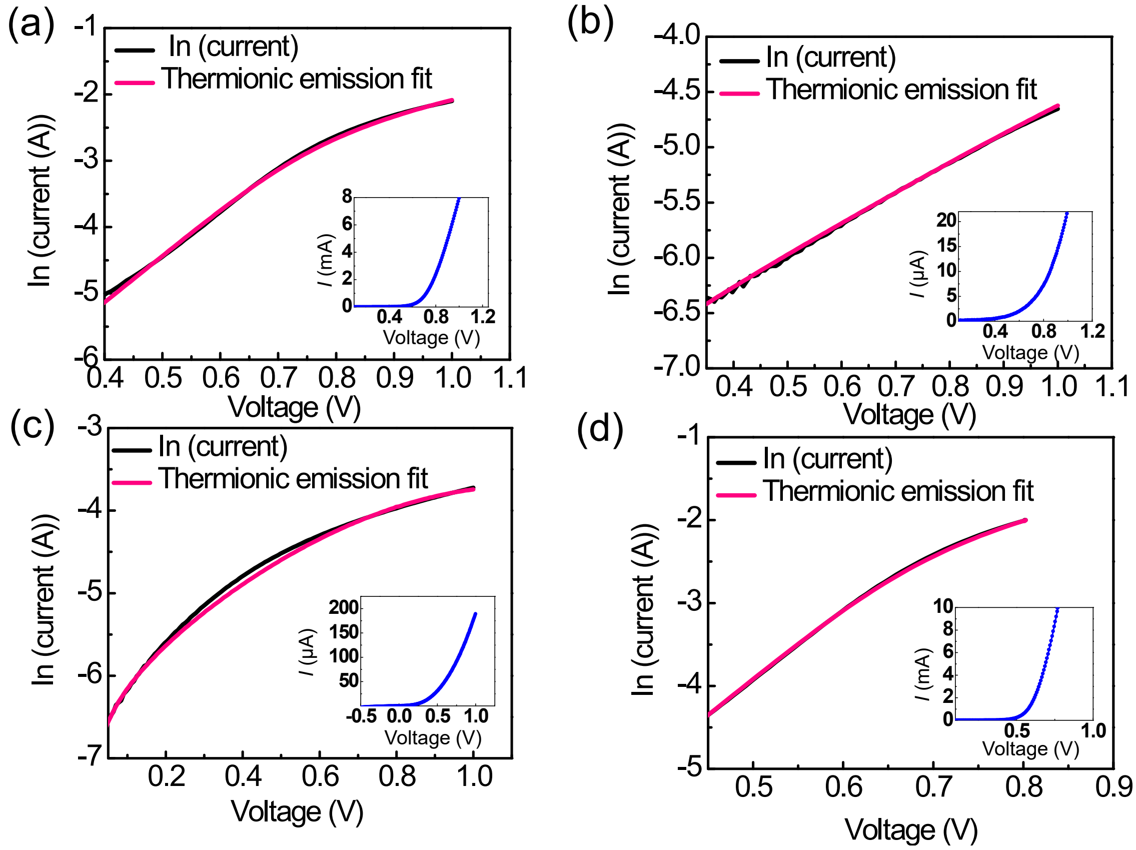

Figure S5. Thermionic emission fitting of the I–V curves to determine the Schottky barrier height (Φ_n) and ideality factor (η) of **(a)** Au/TNA 30, **(b)** Au/TNA 50, **(c)** Ag/TNA 30, and **(d)** Ag/TNA 50 nanodiodes. The insets show the corresponding I–V characteristics of the nanodiodes

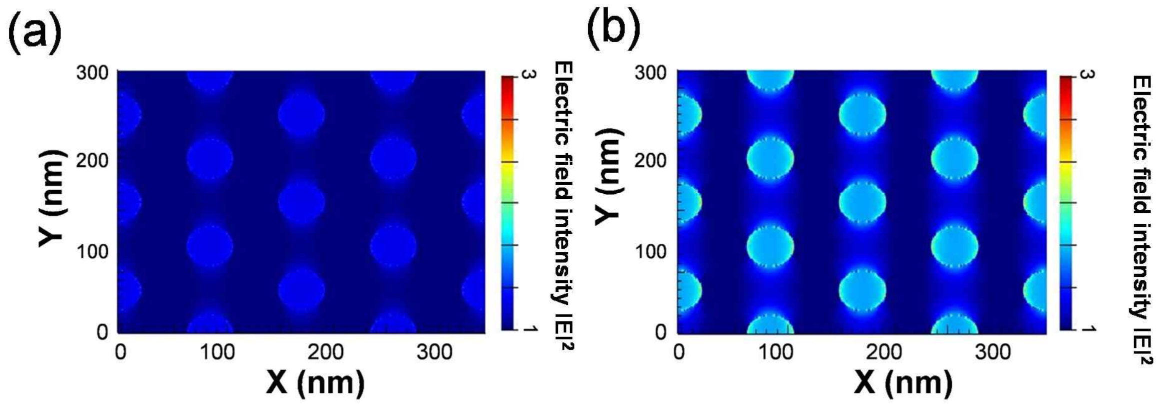

Figure S6. Finite-difference time-domain simulations: Field distribution pattern around the nanostructures of the Au/TNA observed at the extremes of the visible wavelengths of **(a)** 450 nm and **(b)** 750 nm.
